# A Key Major Guideline for Engineering Bioactive Multicomponent Nanofunctionalization for Biomedicine and Other Applications: Fundamental Models Confirmed by Both Direct and Indirect Evidence

**DOI:** 10.1155/2017/2867653

**Published:** 2017-11-29

**Authors:** Roya Dastjerdi, Andreas Scherrieble, Shiva Bahrizadeh, Fatemeh Avareh Sadrabadi, Laleh Hedayat

**Affiliations:** ^1^Textile Engineering Department, Yazd University, Yazd, Iran; ^2^German Institutes of Textile and Fiber Research Denkendorf, Koerschtalstrasse 26, 73770 Denkendorf, Germany; ^3^Microbiological Laboratory, Boomazma Co., Yazd Science and Technology Park, Yazd, Iran

## Abstract

This paper deals with the engineering multicomponent nanofunctionalization process considering fundamental physicochemical features of nanostructures such as surface energy, chemical bonds, and electrostatic interactions. It is pursued by modeling the surface nanopatterning and evaluating the proposed technique and the models. To this end, the effects of surface modifications of nanoclay on surface interactions, orientations, and final features of TiO_2_/Mt nanocolloidal textiles functionalization have been investigated. Various properties of cross-linkable polysiloxanes (XPs) treated samples as well as untreated samples with XPs have been compared to one another. The complete series of samples have been examined in terms of bioactivity and some physical properties, given to provide indirect evidence on the surface nanopatterning. The results disclosed a key role of the selected factors on the final features of treated surfaces. The effects have been thoroughly explained and modeled according to the fundamental physicochemical features. The developed models and associated hypotheses interestingly demonstrated a full agreement with all measured properties and were appreciably confirmed by FESEM evidence (direct evidence). Accordingly, a guideline has been developed to facilitate engineering and optimizing the pre-, main, and post-multicomponent nanofunctionalization procedures in terms of fundamental features of nanostructures and substrates for biomedical applications and other approaches.

## 1. Introduction

A novel approach, after raveling the excellent properties of nanostructures [1–25], is the use of multicomponent formulation of nanostructures [26–33] to achieve outstanding features. Designing a good engineered process to apply different kinds of nanostructures, accompanied with each other, can facilitate achieving the desirable properties. Surface features of biomaterial like roughness, surface electrical charges, wetting behavior, and so forth can determine the cell response and biocompatibility as required in different biomedical approaches [34, 35]. The desired modification in these surface features can be designed by a well-engineered multicomponent nanofunctionalization. However, the critical challenge is finding the key factors and approaches for predicting, engineering, and modeling the multicomponent nanofunctionalization which can be used as a guideline for future designing. Among nanostructures with different shapes and geometries, nanoparticles and nanolayers are the most used species. Among the nanoparticles (NPs), TiO_2_ [36–41], and among the nanolayers (NLs), clay [42–44], are the most reported nanostructures in biomedical approaches as well as other applications. They are the safest nanostructures enjoying remarkable properties and a wide range of applications [45]. Moreover, a certain bioinspired nanoroughness and/or a synergistic effect can be accomplished by an appropriate multicomponent nanopatterning on surface of biomedical membranes or devises.

Given the importance of the aforementioned approaches in the case of multicomponent nanofunctionalization, here, the TiO_2_-containing nanolayered silicate, montmorillonite (Mt), as an isotropic/anisotropic mixed nanocolloid, has been used to achieve multiple-size nanoroughness, as well as multifunctional properties via ultrasound-assistant exhaustion process on textiles, followed by the polysiloxane posttreatment. Above all, a key major guideline on engineering multicomponent nanofunctionalization has been proposed considering fundamental physicochemical features of nanostructures such as surface energy, chemical bonds, and electrostatic interactions. The fundamental models were confirmed by both direct and indirect evidence. By achieving this goal, a guideline has been developed to facilitate engineering the nanofunctionalization procedures of biomaterials and other supplies.

## 2. Experimental Section

### 2.1. Materials

Natural montmorillonite, Cloisite® Na^+^, and organomodified montmorillonite (Cloisite 30B: sodium montmorillonite modified by methyl, tallow, bis-2-hydroxyethyl, quaternary ammonium chloride), supplied by Southern Clay Company, were used to prepare the intercalated nanolayer colloidal solutions. TiO_2_ (P25 nano-titanium dioxide) was kindly provided by Evonik (Degussa) Corporation. TiO_2_ P25 contains 80 wt% anatase and 20 wt% rutile structures and has 25–30 nm particle size [46]. Polysiloxane CT 208 E emulsion was kindly provided by Wacker Finish. CT 208 E is a water-based polysiloxane emulsion, a commercially available product, by Wacker Finish (Germany). It contains 66% solid content containing 60% active substance content of aminofunctional polysiloxane, as stated by supplier.

### 2.2. Methods

At first, colloidal solutions of 0.1 wt% natural or organomodified clay nanolayers (NLs or ONLs) have been prepared using magnetic stirrer and ultrasonic waves. Then, keeping the dispersing powers, the TiO_2_ nanoparticles (NPs, 0.1 wt%) have been gradually added to the clay colloidal solutions. The cotton fabrics have been treated via exhaustion process under ultrasonic waves for 45 min. Some samples have been also designed applying only NLs, ONLs, or NPs, to compare the effect of each component. The produced samples have been coded by their treatment components as NL, ONL, NP, NL/NP, and ONL/NP (Table 1). All the production steps have been performed at ambient temperature and atmosphere. The posttreatment by XPs was performed as follows. Each sample was immersed in 2 wt% polysiloxane solution for 5 s at ambient temperature and atmosphere and squeezed by pad to 100% wet pick-up. Then, the padded samples were dried at 100°C. The process has been performed on mercerized and unmercerized cotton fabrics with the same structures to investigate the pretreatment effect. The mercerized samples were indicated by adding “M.” prior to their code.

### 2.3. Characterizations

#### 2.3.1. Hydrophobicity

The hydrophobicity of samples was studied, according to AATCC Standard Test Method 79, by measuring the time required for water droplet to be completely spread on the fabric surfaces. To this end, water was dropped from 1 cm on the fabric surface by a small syringe. The time of the complete absorption of 10 *μ*l water droplets on the fabric surfaces (in 27°C and 30% relative humidity) was measured for 10 replicates after which the average value was reported. Contact angle (CA) of a 10 *μ*l distilled water droplet dropped from a distance of 1 cm was measured 5 seconds after placing on the fabric surface using a self-developed goniometer apparatus equipped with a high resolution camera and suitable lens. The volume of the droplets was exactly controlled using a special capillary connected to an Atom Syringe Pump S-1235, Japan. The average of contact angles of two sides of 6 drops, determined by using MB-Ruler software, was calculated and reported as CA of each sample [47].

#### 2.3.2. Scanning Electron Microscopy

Field emission scanning electron microscope (FESEM) micrographs were obtained using a Vega/Tescan, Mira 3-XMU field emission scanning electron microscope with both low and high magnifications. The samples were coated by a thin layer of gold, sputtered on the samples with a thickness of about 30 nm, before FESEM analysis.

#### 2.3.3. Stiffness

The Shirley stiffness test was used as a criterion for stiffness and the bending length was reported for test specimens cut in 25 mm width and 200 mm length parallel to the warp. This experiment was performed for all samples with 6 replicates and the average values were reported.

#### 2.3.4. Crease Recovery Angle

The wrinkle recovery angle in the warp and weft fabric directions was measured according to BS 3086: 1972 test method by the Shirley Crease Recovery tester, with 4 replicates, and the average values were reported.

#### 2.3.5. UV Protection Properties

Evaluating the color changes of wool fabrics dyed by a highly UV sensitive dye (0.5 wt% Methylene Blue) which were covered by different modified and unmodified samples under UV irradiation was considered as a criterion for the comparison of the UV protection properties of samples [48]. UV irradiations were provided by a Philips Cleo UV lamp HPA 400S located 25 cm above the covered samples for 18 h. After UV irradiation, the color changes were studied based on reflectance data using an X-rite spectrophotometer according to the formula reported in our previous paper [48].

#### 2.3.6. Photocatalytic Bioactivity

Bioactivity of the functionalized samples was investigated on the five-time-washed samples via methods exactly reported in our previous papers [48] but with some modifications as follows. The photo-irradiation was conducted by a conventional fluorescent lamp (20 W Sinar® Merbabu, Indonesia) located 60 cm above the samples for 18 hours.* R* (%) is the percentage of reduction of bacteria as compared to the untreated sample under the same test conditions, referring to the antibacterial efficiency. The washing procedure on samples was performed using a Rotawash Model M228B Launderometer according to AATCC test method 61(2A)-1996 to 5-cycle washing.

## 3. Results and Discussions

### 3.1. Hydrophobicity and Stain Repellency

In recent years, developing a bioinspired nanoroughness to achieve the demanded hydrophobicity or hydrophilicity is increasingly investigated [49, 50]. Wetting behavior of biomedical devices is one of the most important criteria indicating their performance for different applications [51–53]. For instance, an ultra-hydrophilic feature is vital for haemostatic and clotting membranes [54]. Superhydrophobic medical devices can be designed for many other cardiovascular applications such as pericardial substitutes [52], artificial vessels, and stents. Consequently, the appropriate feature must be engineered for each application utilizing innovative techniques.

**Figure 1:**
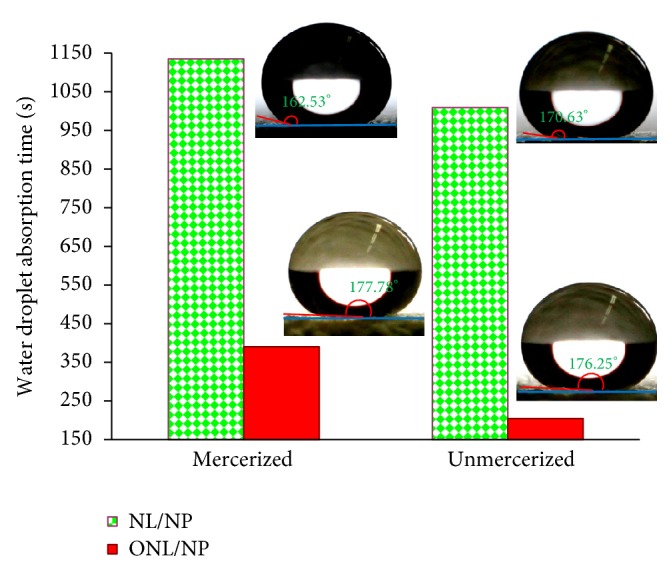
The effect of pretreatment (mercerizing) and nanolayer modification on hydrophobicity of XPs-treated samples.

**Figure 2:**
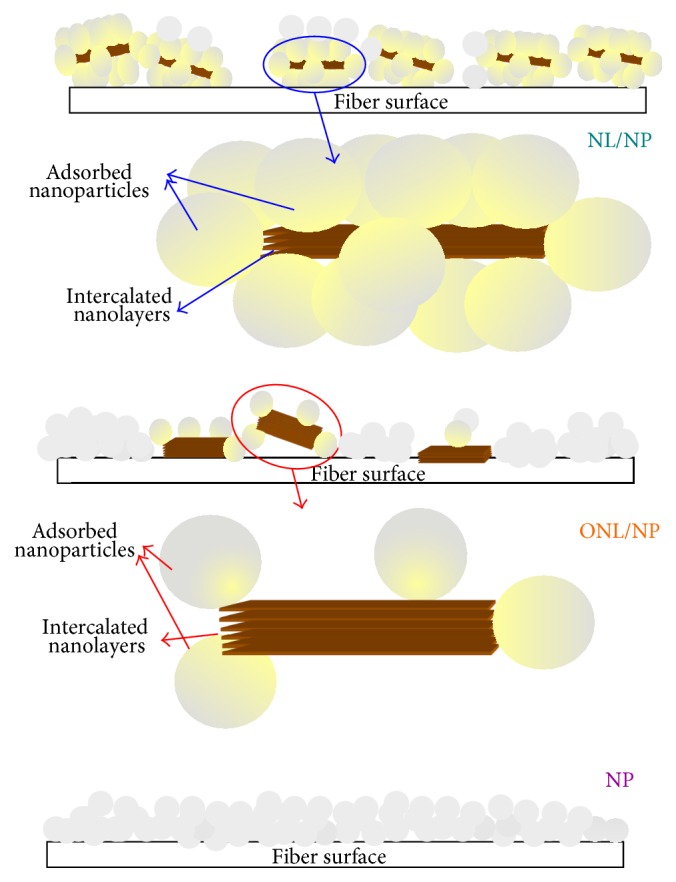
Schematic model for surface orientation of nanostructures on nanolayer/nanoparticle (NL/NP), organomodified nanolayer/nanoparticle (ONL/NP), and nanoparticle (NP) functionalized samples.

**Figure 3:**
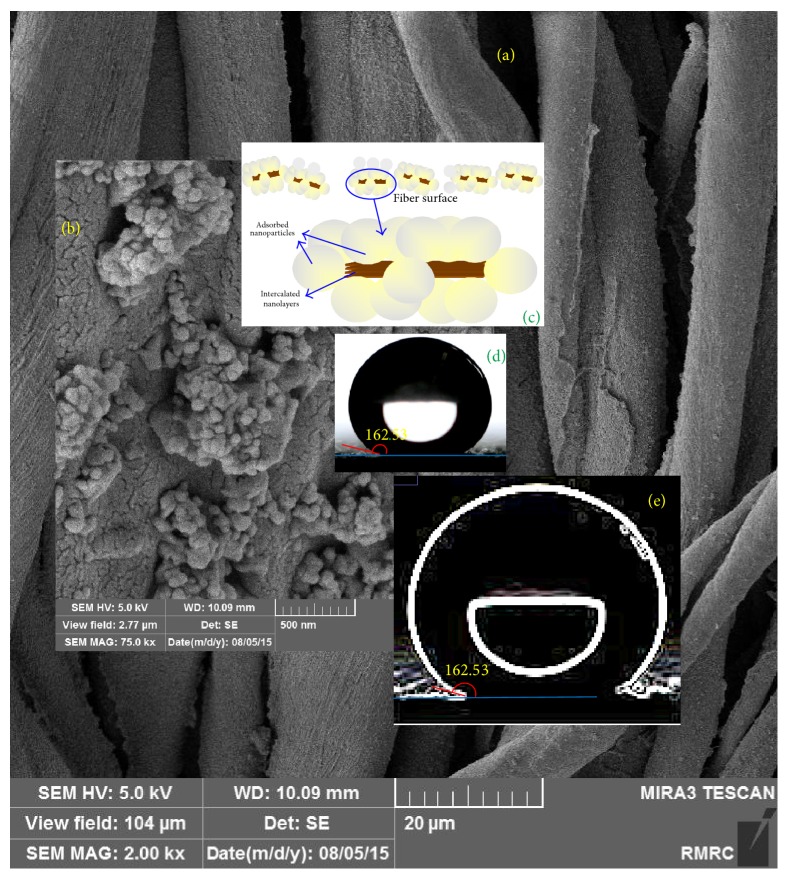
((a) and (b)) FESEM micrographs of fiber surfaces functionalized by nanocolloidal natural montmorillonite and TiO_2_ (NL/NP), with low and high magnification, respectively, (c) schematic model proposed according to the fundamental physic-chemical phenomena for this sample (NL/NP) in comparison with the microscopic outcomes, (d) water droplet image and contact angle (°) on this sample, and (e) image “(d)” processed by “the find edge option” of ImageJ software to clarify of the droplet boundaries for precise evaluation of contact angle.

**Figure 4:**
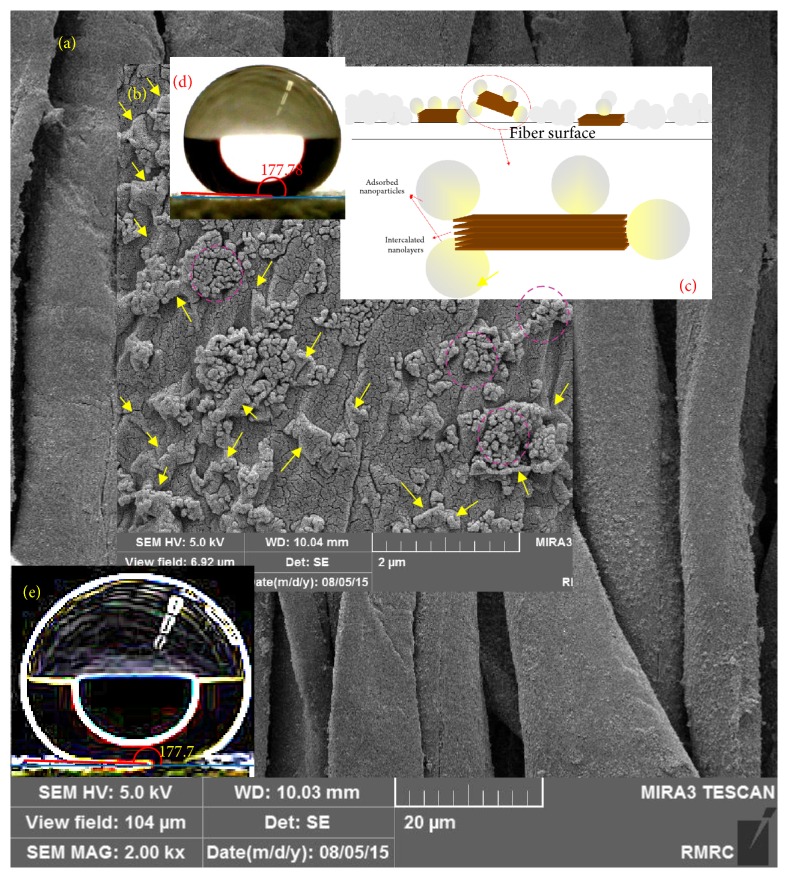
((a) and (b)) FESEM micrographs of fiber surfaces functionalized by nanocolloidal organomodified montmorillonite and TiO_2_ (ONL/NP), with low and high magnification, respectively; arrows point out separated nanolayers and circles indicate nanoparticles, (c) schematic model proposed according to the fundamental physic-chemical phenomena for this sample (ONL/NP) in comparison with the microscopic outcomes, (d) water droplet image and contact angle (°) on this sample, and (e) image “(d)” processed by “the find edge option” of ImageJ software to clarify the droplet boundaries for precise evaluation of contact angle.

**Figure 5:**
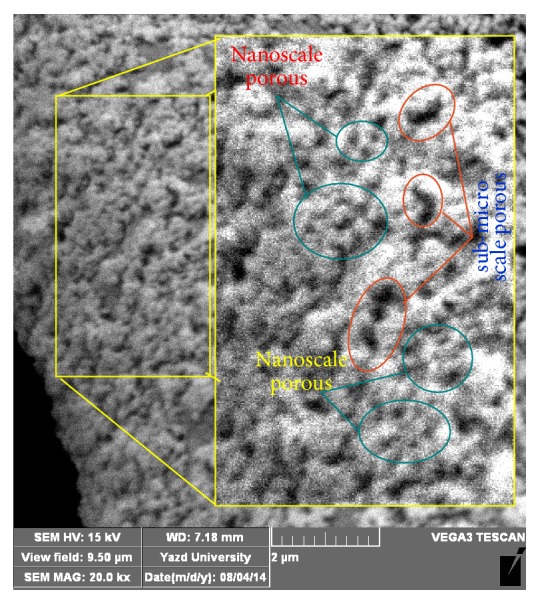
SEM micrographs of fiber surfaces functionalized by nanocolloidal TiO_2_ creating multiscale nano/submicroporous interfaces.

Wettability of samples treated with nanostructures and XPs can be considered as a criterion indicating the formation of a complete layer of XPs on the nanostructures, promising ideal fastness as well as multifunctional properties [48]. In fact, the maximum hydrophobicity can be accomplished by forming a complete layer of XPs. The incomplete layer leads to a reduced hydrophobicity. On the other hand, extra XPs, more than the amount required to form one layer, will reduce the hydrophobicity due to inverse orientation of XPs hydrophilic groups on the first layer. On this basis, this measurement is the most important feature to provide certain information required to determine the appropriate chemical ratios of nanostructures and XPs. Consequently, in a predesigned experiment, the proper XPs ratio has been determined for the nanoparticle-containing intercalated nanolayers produced in this research. According to the unreported results, the best XPs concentration, which reached 2 wt%, was derived and used in this research. The average value of water droplet absorption time for XPs-treated nanofunctionalized samples has been reported in Table 2. The water droplet absorption time for unmodified cotton fabrics was less than one second. Water droplets were also fast spread on all nanofunctionalized samples without XPs treatment. The cross-linkable polysiloxane increased water droplet absorption times on all nanofunctionalized samples (Table 2). The hydrophobic properties achieved by colloidal solution of TiO_2_ containing nanolayers were more than those achieved by colloidal solution of pure nanolayers without NPs. This result was recorded for both kinds of nanolayers. Generally, creating hydrophobic nanoroughness can increase the hydrophobic properties of surfaces. As mentioned above, XPs coverage with a suitable concentration can provide the hydrophobic feature on the nanofunctionalized samples. Fine nanoroughness can provide more and finer air pocket and increase hydrophobicity [55]. The nanoroughness created by TiO_2_ nanopatterns on nanolayer was more effective than nanoroughness created by pure nanolayers. The effects of nanoclay types on hydrophobicity of nano-TiO_2_/clay functionalized samples have been compared with each other as shown in Figure 1. Unexpectedly, the hydrophobicity created by TiO_2_ NPs containing natural montmorillonite was significantly more than that of TiO_2_ containing organomodified montmorillonite. To explain this phenomenon, we should thoroughly consider the whole process. In this research, TiO_2_/clay nanocolloidal solutions have been prepared by gradually adding TiO_2_ NPs to clay nanocolloidal solutions under ultrasound. Since the difference of surface energy levels between natural montmorillonite and TiO_2_ NPs is less than that of organomodified montmorillonite and TiO_2_ NPs, the affinity of nanoparticles to be absorbed on the natural montmorillonite is more than that of the organomodified layers in the colloidal solution. This would be mainly intensified by electrostatic interactions between natural montmorillonite and TiO_2_ NPs due to the negative surface charge of TiO_2_ nanoparticles above pH 5.6 (here the solvent was distilled water with pH about 7) and the positive charge of Na^+^ ions on natural montmorillonite, Cloisite Na^+^. Consequently, the colloidal solution of natural montmorillonite and TiO_2_ NPs can create an intensified nanoroughness on the functionalized fabrics (NL/NP) as shown schematically in Figure 2. Creating this intensified, finer, and more regular nanoroughness can enhance hydrophobicity following the XPs treatment, while nanoparticles and nanolayers are mostly separated in the colloidal solution of organomodified montmorillonite and TiO_2_ NPs (ONL/NP). Moreover, because of the enhanced affinity of organomodified layers and cotton fiber surfaces, the separated layers are attracted to lie on the fabric surfaces as a result of the hydrophobic-hydrophobic interactions among the organic groups on layers and fiber surfaces, as well as potentiality to form hydrogen bonds between their OH groups. Therefore, their cooperation to create nanoroughness reduced as compared to natural montmorillonite. These hypotheses have been interestingly confirmed by microscopic evidence. The FESEM micrographs have been demonstrated in Figures 3 and 4. The water droplet absorption time for XPs-treated mercerized samples was more than that of unmercerized samples. This pointed out the effect of this conventional pretreatment on increasing the reactivity intensifying of adsorption of nanostructures during the nanofunctionalization process. As is well known, mercerizing causes some structural changes in cotton fibers. The reduction of crystallinity as a result of mercerizing leads to the increase of the amorphous regions which have a good potentiality for the adsorption of nanostructures. Increasing fibrous porosity, moisture regains, fabric hygroscopicity, absorbency, smoother morphology, and so forth caused by this pretreatment “mercerizing” [56, 57] can direct the better adsorption of nanostructures on mercerized fabrics. The maximum hydrophobicity has been obtained for XPs-treated mercerized sample functionalized with only TiO_2_ NPs. Unexpectedly, the enhanced hydrophobicity was reduced by adding nanolayers to nanoparticles. This can be explained by reducing the created nanoroughness due to covering some parts of nanoparticle roughness by nanolayers. Moreover, the created roughness in the absence of layers is finer and more regular (see Figure 5), providing smaller air pockets on the interface of fabric and droplets which require a higher level of energy to be overcome [55]. However, all XPs-treated nanofunctionalized samples demonstrate significant increases of absorption times as compared to both untreated mercerized and unmercerized cotton. This enhanced hydrophobicity along with a high contact angle (about 150°, see Table 2) provided a superhydrophobic feature on cotton fabrics (Figure 3). The achieved hydrophobicity satisfied the perfect stain repellency. XPs-treated samples can quickly repel and remove the stain droplets before they can be adsorbed, while the stain droplets were completely absorbed on the untreated samples. It should be pointed out that achieving such high contact angle on the textiles substrates is absolutely more difficult than other substrate like glass, plastic sheets or films, steel or other metallic devices or surfaces, and so forth. Numerous research works have also reported achieving a superhydrophobicity using fluorinated compounds and/or with a smaller droplet [58–62]. A recently published paper which has been also considered as a cutting edged publication [63] has yet used organic solvents to apply a fluorine-free treatment to textiles. Moreover, none of its droplet images [63] shows a contact angle bigger than our achievements, even with a smaller droplet [63]. We are reporting a water-based fluorine-free superhydrophobic treatment on cotton fabrics in this paper. The droplet images recorded in our research point to the achievement of a unique contact angle which has not been reported so far either on textiles or on other substrates. For example, the droplet on Figure 4(d) and specially its sharp-edge version (Figure 4(e)), processed by “*the find edge option*” of* ImageJ software* to clarify of the droplet boundaries and more precise evaluation, show achieving a contact angle of about 177°. However, we have relinquished this evidence and prudently reported more than 150° for the cases which were more than 150° (Table 2), while they (CAs) were notably more for all of the droplet images (see also Figures 1 and 3). Therefore, this treatment can be suggested for developing blood-repellent hospital textiles [64], as well as hygienic home and industrial textiles. The superhydrophobic samples have also a remarkable potentiality for antiadhesive biomedical applications like pericardial substitutes. The achievements in this paper meet the sharpest cutting edged scientific subjects [65].

### 3.2. Field Emission Scanning Electron Microscopy (FESEM)

Field emission scanning electron microscopy (FESEM) was used to study the surface orientation of nanostructures on nanocolloidal functionalized samples. Figures 3(a) and 3(b) show FESEM micrographs of fiber surfaces functionalized by nanocolloidal natural montmorillonite and TiO_2_. Similarly, Figures 4(a) and 4(b) demonstrate the evidence of organomodified montmorillonite and TiO_2_. Although cotton fibers naturally pose an uneven morphology, the micrographs with low magnifications, x = 2000, shown as background (Figures 3(a) and 4(a)), appreciably corroborated forming an even coating of nanostructures with a good dispersibility on each sample. Figures 3(a) and 4(a) are also implying formation of a thin even coverage of nanomaterials around the fibers surfaces without blocking the fabric holes. In fact, since the size of fabric holes are considerably bigger than the thickness of the formed thin coverage, the fabrics air permeability is not negatively affected by such even treatment as the previous report [48] also confirms this issue. The appropriate magnifications have been also provided to show the surface orientation of nanostructures for each sample. The given evidence by FESEM surprisingly confirmed all developed models (Figures 2, 3(c), and 4(c)) and associated theories raised based on the wetting properties. As thoroughly discussed in Section 3.1, these models and theories have been yielded according to the results along with considering fundamental features of interfaces, consisting of the differences of surface energy levels between nanostructures, surface charges, and surface interactions of the nanolayers and nanoparticles, as well as their interactions and orientations on textile surfaces. Wonderfully, all achievements are also in complete agreement with all other characteristics discussed in the following sections.

### 3.3. UV Protection Properties

**Figure 6:**
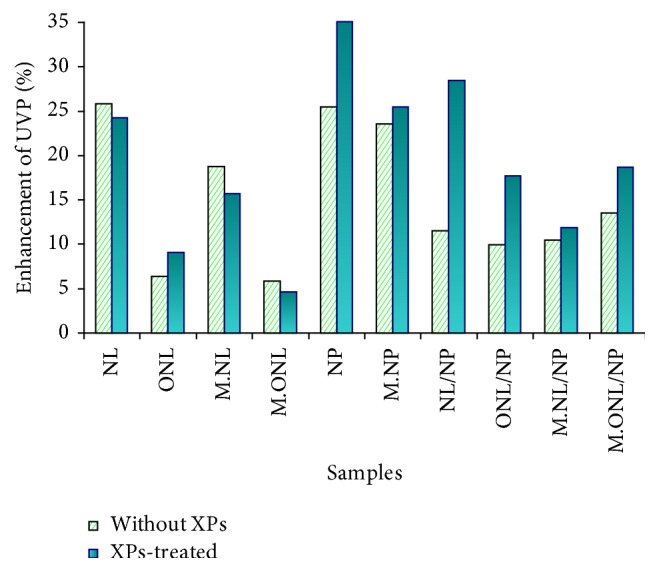
Enhancement of UV protection properties (%) as compared to control samples (C and MC).

The results showed that the enhancement of UV protection properties of fabrics functionalized by TiO_2_ containing organomodified montmorillonite was more than TiO_2_ containing natural montmorillonite (Figure 6). This implies the intensified cocovering effect of natural nanolayers and nanoparticles due to their electrostatic interactions as thoroughly discussed in Section 3.1, schematically shown in Figure 2, and confirmed by microscopic evidence (Figures 3 and 4).

In addition, as mentioned before, organomodified montmorillonite is attracted to lie on the fabric surfaces due to the hydrophobic-hydrophobic interactions and hydrogenic bonds between the organic groups on layers and fiber surfaces, and in this way, they can provide better support for fiber (see scheme in Figures 2 and 4). This result interestingly confirmed the developed models on the basis of wettability of samples. The enhancement of UV protection properties is also more for TiO_2_ treated fabrics than that of TiO_2_ containing nanolayers indicating the cocovering effect of nanolayers and nanoparticles, in agreement with the developed models and results of measuring hydrophobicity. UV protection properties were positively affected by XPs treatment. In fact, XPs treatment can improve the UV protection properties on nanofunctionalized surfaces via covering certain parts of bigger nanoroughness followed by reducing irregular multi-internal reflections [48], increasing the reflection proportion against light transfer [47], inducing light to the TiO_2_ and clay nanostructures as the UV-absorbent, and increasing the absorption proportion against light transfer.

### 3.4. Fabric Stiffness

Functionalization of fabric surfaces with inorganic nanostructures can commonly lead to the decrease of fabric softness [48]. Bending length is one of the best indices to decide on the fabric softness. The results showed that the values of bending length of mercerized nanofunctionalized fabrics are generally more than those of unmercerized ones. This confirmed the more reactivity and better adsorption of nanostructures on mercerized cotton, interestingly in agreement with the results and discussions developed in Section 3.1. However, XPs, as a softener, have also compensated for the reduction of fabric softness and reduced the bending length (Table 3). Therefore, XPs treatment can promise more comfortable multifunctional textiles via improving softness and handle.

### 3.5. Anti-Crease Properties

The recovery of wrinkle has been reduced by nanofunctionalization due to steric hindrance of inorganic nanostructures against the movement of polymeric chains (Table 3). However, XPs treatment has compensated for this effect and improved antiwrinkle properties of XPs-treated nanofunctionalized fabrics due to its softening as well as plasticizing effects. Moreover, the formation of network-like layer of polysiloxane on the fabric surfaces [47] can increase their resistance to crease.

### 3.6. Photo-Bioactivity

The 5-time-washed XPs-treated samples with the best multifunctional properties were chosen for evaluation of their antibacterial activities against* Escherichia coli* (ATCC 25922). The concentration of the bacteria in the primary inoculums was 2.1 × 10^6^. The number of bacteria recovered from the inoculated untreated sample immediately after inoculation was estimated as 3.7 × 10^5^ cfu·ml^−1^. The average number of bacteria recovered from the incubated untreated samples under the photo (81 × 10^5^ cfu·ml^−1^) was considered as a reference to calculate the antibacterial activity.

**Figure 7:**
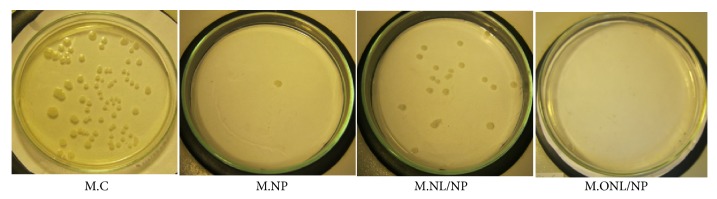
Qualitative comparing photo-bioactivity basis of 10^5^ times diluted bacteria, recovered from the inoculated test specimen swatches of washed XPs-treated samples.

Table 4 reports the antibacterial activity of the tested samples. Sufficient antibacterial efficiency was achieved by the mercerized organomodified montmorillonite and TiO_2_-treated sample (M.ONL/NP) as well as the mercerized TiO_2_-treated sample (M.NP). Although the antibacterial activity of the mercerized sample treated with Cloisite Na^+^ and TiO_2_ (M.NL/NP) was also satisfying for many applications, it could not compete with the best sample. This can be caused by cocovering of nanostructures as thoroughly discussed before. The results of the designed qualitative test for comparing the antibacterial efficiency of some samples are also exhibited in Figure 7.

The antibacterial activity, along with other practical features of treated samples, as discussed above, makes them an excellent suggestion for hospital textiles [66–68] as well as many hygienic garments and home textiles.

## 4. Conclusions

A guideline to realize the key factors and approaches for predicting, engineering, and modeling the multicomponent nanofunctionalization for biomedical devices or other application has been developed in this research. Moreover, a novel bioactive polysiloxane-shield nanolayer patterned by nanoparticles has been produced and investigated. The key role of the surface properties of applied nanostructures on the surface patterning and multifunctional properties of treated surfaces has been discussed and modeled according to their surface energy, chemical bonds, and electrostatic interactions. The accomplished models and associated hypotheses have appreciably a full agreement with all the investigated characteristics and have been interestingly confirmed by microscopic evidence. The results and models developed in this paper serve as a major guideline in the following highly interesting topics:

- Engineering the pre-, main, and post-multicomponent nanofunctionalization procedures in terms of fundamental features of nanostructures and different surfaces
- Designing novel multicomponent nanocolloids, nanocoatings, and nanocomposites for biological and/or multifunctional approaches
- Developing discussions and models for multifunctional features of modifications based on multicomponent nanostructures with different geometries including isotropic and anisotropic structures
- Achieving multifunctional properties by polysiloxane-shield nanoparticle-containing intercalated nanolayers on different surfaces
- Comparing polysiloxane-shield nanolayer and nanoparticle functionalizations
- Studying the effect of nanolayer surface modifications on the properties of functionalized surfaces
- Comparing the effect of functionalization with nanolayers, nanoparticles, and nanoparticle-containing intercalated nanolayers
- Studying the effect of surface modifications of nanolayers on their patterning by nanoparticles through their interactions, orientations, and so forth
- Investigating the effect of polysiloxane posttreatment in all aforementioned topics via comparing XPs-treated samples with samples without XPs treatment
- Studying the effect of substrate pretreatment on all aforementioned functionalizations.

## Supplementary Material

I M. NP.Ps.avi: This movie (Movie I) shows the stain- repellency of XPs treated mercerized sample functionalized by nanoparticles. Nanofunctionalized samples treated with cross-linkable polysiloxane (XPs) can quickly repel and remove the stain droplets before they can be adsorbed (see Movie I-III); while the stain droplets were completely absorbed on the untreated sample (see Movie IV).
II M.NL.NP.Ps.avi: This movie (Movie II) shows the stain- repellency of XPs treated mercerized sample functionalized by nanoparticle-containing intercalated nanolayers. Nanofunctionalized samples treated with cross-linkable polysiloxane (XPs) can quickly repel and remove the stain droplets before they can be adsorbed (see Movie I-III); while the stain droplets were completely absorbed on the untreated sample (see Movie IV).
III M.ONL.NP.Ps.avi: This movie (Movie III) shows the stain- repellency of XPs treated mercerized sample functionalized by nanoparticle-containing intercalated organo-modified nanolayers. Nanofunctionalized samples treated with cross-linkable polysiloxane (XPs) can quickly repel and remove the stain droplets before they can be adsorbed (see Movie I-III); while the stain droplets were completely absorbed on the untreated sample (see Movie IV).
IV C.avi: This movie (Movie IV) shows the complete absorption of a stain droplet on the untreated sample. Nanofunctionalized samples treated with cross-linkable polysiloxane (XPs) can quickly repel and remove the stain droplets before they can be adsorbed (see Movie I-III); while the stain droplets were completely absorbed on the untreated sample (see Movie IV).
Graphical Abstract.pdf: Modeling of surface orientation of nanostructures on the fabric surfaces, proposed according to the fundamental physic-chemical phenomena, confirmed by the microscopic outcomes.

## Figures and Tables

**Table 1:** Introducing different samples.

Sample code	Pretreatment	Nanolayer functionalization		Nanoparticle functionalization
	(Mercerizing)	Natural NLs	Organomodified NLs	TiO_2_ NPs
C	*✗*	*✗*	*✗*	*✗*
M.C	✓	*✗*	*✗*	*✗*
NL	*✗*	✓	*✗*	*✗*
ONL	*✗*	*✗*	✓	*✗*
M.NL	✓	✓	*✗*	*✗*
M.ONL	✓	*✗*	✓	*✗*
NP	*✗*	*✗*	*✗*	✓
M.NP	✓	*✗*	*✗*	✓
NL/NP	*✗*	✓	*✗*	✓
ONL/NP	*✗*	*✗*	✓	✓
M.NL/NP	✓	✓	*✗*	✓
M.ONL/NP	✓	*✗*	✓	✓

**Table 2:** Wetting properties of XPs-treated nanofunctionalized samples.

Sample code	Water droplet absorption time (s)	Water droplet contact angle (°)
NL	205	149.8 ± 1.2
ONL	118	149.8 ± 1.8
M.NL	134	150.2 ± 1.5
M.ONL	197	148.1 ± 2.1
NP	251	148.6 ± 1.3
M.NP	2068	>150 ± 2.5
NL/NP	1009	>150 ± 2.5
ONL/NP	204	>150 ± 2.5
M.NL/NP	1135	>150 ± 2.4
M.ONL/NP	390	>150 ± 2.5

**Table 3:** Bending length and crease recovery angle of different samples.

Sample code	Bending length (cm)		Crease recovery angle (warp) (°)		Crease recovery angle (weft) (°)	
	Before XPs treatment	After XPs treatment	Before XPs treatment	After XPs treatment	Before XPs treatment	After XPs treatment
C	5.4	5.7	121	134	122	131
M.C	6.2	5.7	135	131	120	126
NL	5.6	5.4	101	122	111	128
ONL	5.3	5.3	107	132	109	126
M.NL	6.3	5.6	115	126	109	134
M.ONL	6.2	5.8	116	118	120	134
NP	5.2	5.4	110	128	119	126
M.NP	6.4	5.7	103	115	105	128
NL/NP	4.2	5.2	105	10	107	116
ONL/NP	5.5	5.1	108	130	114	116
M.NL/NP	6.3	5.8	109	124	113	121
M.ONL/NP	6.1	5.6	116	120	103	123

**Table 4:** Bioactivity of washed X-Ps treated samples.

Samples	Bacteria number ×10^−5^^*∗*^	*R*%
MC	81	0.00
M.NP	1	98.77
M.NL/NP	19.56	75.85
M.ONL/NP	0.44	99.46

^*∗*^The average number of 10^5^ times diluted bacteria recovered from the inoculated test specimen swatches.
