# Supplementary material for: A Key Major Guideline for Engineering Bioactive Multicomponent Nanofunctionalization for Biomedicine and Other Applications: Fundamental Models Confirmed by Both Direct and Indirect Evidence
Source: Biomed Res Int. 2017 Nov 29;2017:2867653. doi: 10.1155/2017/2867653 (PMC5733208; doi:10.1155/2017/2867653)

Graphical Abstract

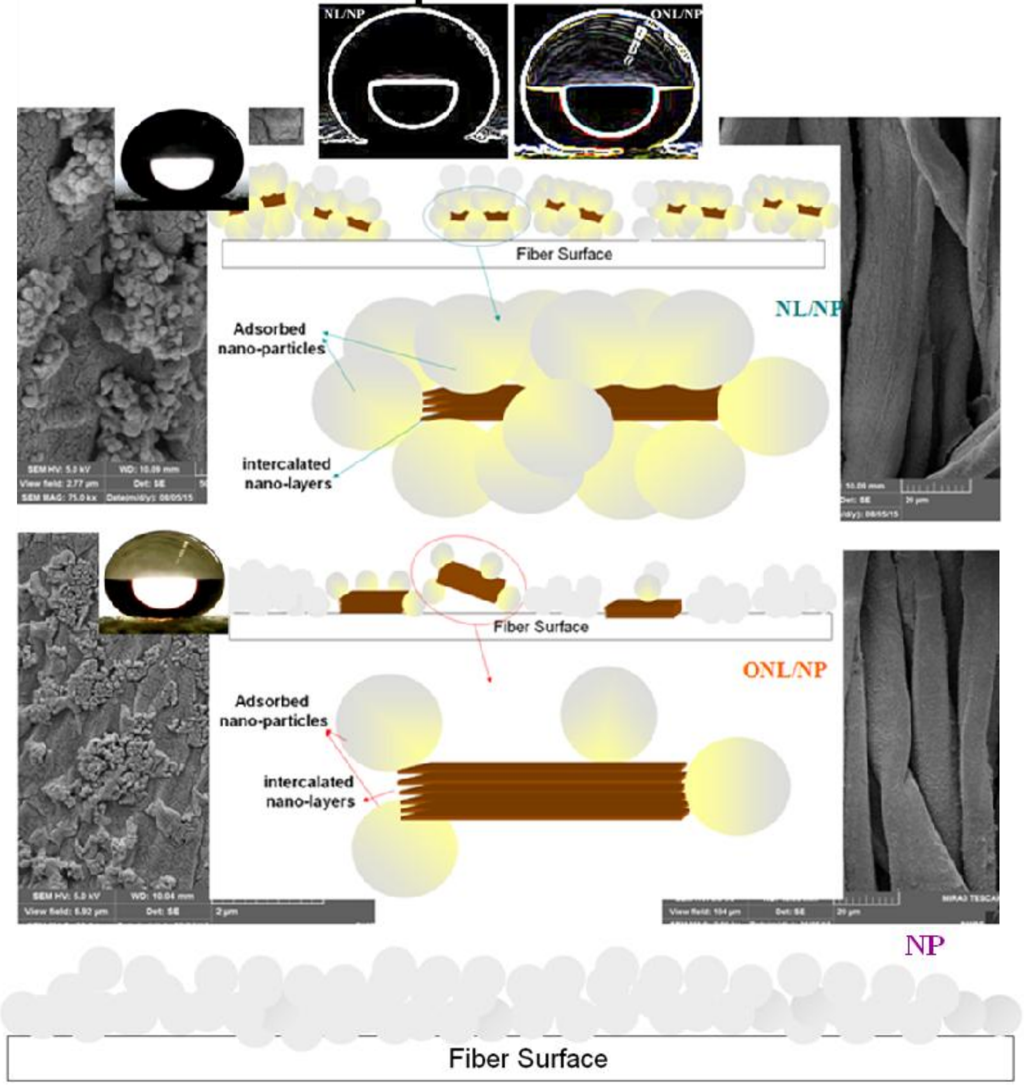

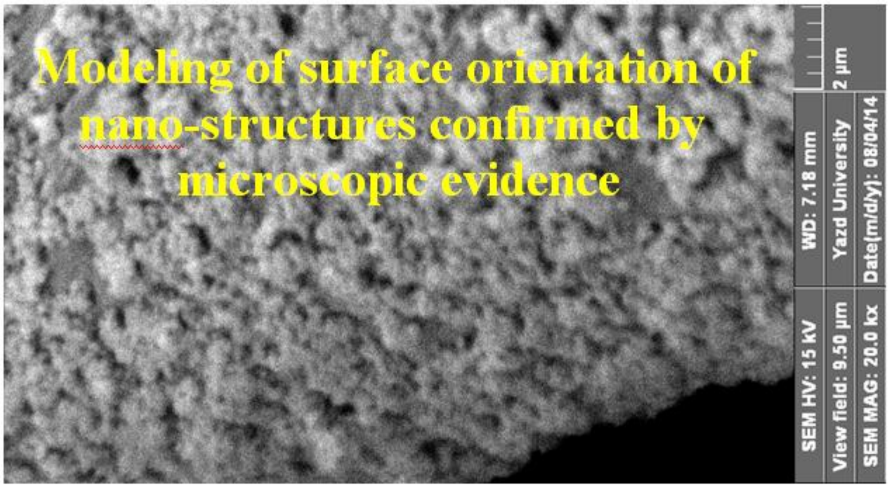

Supplement: Supplementary file 1 — I M. NP.Ps.avi: This movie (Movie I) shows the stain- repellency of XPs treated mercerized sample functionalized by nanoparticles. Nanofunctionalized samples treated with cross-linkable polysiloxane (XPs) can quickly repel and remove the stain droplets before they can be adsorbed (see Movie I-III); while the stain droplets were completely absorbed on the untreated sample (see Movie IV). II M.NL.NP.Ps.avi: This movie (Movie II) shows the stain- repellency of XPs treated mercerized sample functionalized by nanoparticle-containing intercalated nanolayers. Nanofunctionalized samples treated with cross-linkable polysiloxane (XPs) can quickly repel and remove the stain droplets before they can be adsorbed (see Movie I-III); while the stain droplets were completely absorbed on the untreated sample (see Movie IV). III M.ONL.NP.Ps.avi: This movie (Movie III) shows the stain- repellency of XPs treated mercerized sample functionalized by nanoparticle-containing intercalated organo-modified nanolayers. Nanofunctionalized samples treated with cross-linkable polysiloxane (XPs) can quickly repel and remove the stain droplets before they can be adsorbed (see Movie I-III); while the stain droplets were completely absorbed on the untreated sample (see Movie IV). IV C.avi: This movie (Movie IV) shows the complete absorption of a stain droplet on the untreated sample. Nanofunctionalized samples treated with cross-linkable polysiloxane (XPs) can quickly repel and remove the stain droplets before they can be adsorbed (see Movie I-III); while the stain droplets were completely absorbed on the untreated sample (see Movie IV). Graphical Abstract.pdf: Modeling of surface orientation of nanostructures on the fabric surfaces, proposed according to the fundamental physic-chemical phenomena, confirmed by the microscopic outcomes. [file 2867653.f1.zip › Supplementary materials/Graphical Abstract.pdf]
